# Management of traumatic scalp arteriovenous fistula: Case report and literature review

**DOI:** 10.1097/MD.0000000000039764

**Published:** 2024-09-20

**Authors:** Yi-Ying Hsieh, Ching-Chang Chen, Po-Hsun Tu, Shun-Tai Yang, Zhuo-Hao Liu

**Affiliations:** a Department of Neurosurgery, Chang Gung Memorial Hospital at Linkou, Chang Gung Medical College and University, Taoyuan, Taiwan; b Department of Neurosurgery, Graduate Institute of Medical Sciences, College of Medicine, Taipei Medical University, Taipei, Taiwan; c Department of Neurosurgery, Shuang Ho Hospital, Taipei Medical University, Taipei, Taiwan; d Department of Surgery, School of Medicine, College of Medicine, Taipei Medical University, Taipei, Taiwan; e School of Medicine, National Tsing Hua University, Hsinchu, Taiwan.

**Keywords:** head injury, recurrence, scalp arteriovenous fistula, surgical excision, trans-arterial embolization

## Abstract

**Rationale:**

Blunt traumatic arteriovenous fistula (AVF) of scalp, are uncommon and most of them can be secured by simple embolization or surgical ligation of the feeders. Our goal in writing this paper is to show patients with traumatic scalp AVFs how to prevent complications and the likelihood of recurrence.

**Patient concerns:**

Complete treatment and reduce the recurrence rate of traumatic AVF on the scalp.

**Diagnoses:**

Traumatic scalp AVF.

**Interventions:**

Transarterial embolization and surgical resection.

**Outcomes:**

Complete resection of the AVF and subsequent angiography showed resolution of the contralateral lesion.

**Lessons:**

Combination of endovascular embolization with subsequent surgical removal may reduce intraoperative blood loss compared with surgery alone. In addition, a well-designed scalp flap can be performed based on the angiography findings after embolization.

## 1. Introduction

Arteriovenous fistulas (AVFs) of the scalp are direct links between the arterial feeding vessels and the draining veins, without an intervening capillary bed. The causes of scalp arteriovenous fistula (SAVF) include idiopathic, trauma, hair transplantation, and iatrogenic injury. This article describes such a case resulting from blunt head trauma which was treated successfully by complete surgical excision with the aid of preoperative endovascular embolization.

## 2. Case report

A 27-year-old male was referred to our institution for evaluation of a progressively enlarged mass in right temporal region noted since his traffic accident 2 years ago. In addition, pulsatile bruit and dyspnea on exertion were also complained. There is no iatrogenic cause for this patient. Physical examination revealed with a pulsatile subcutaneous mass approximately 4 cm in diameter was noted. Vessels below his temporal scalp were dilated and tortuous.

**Figure 1.**
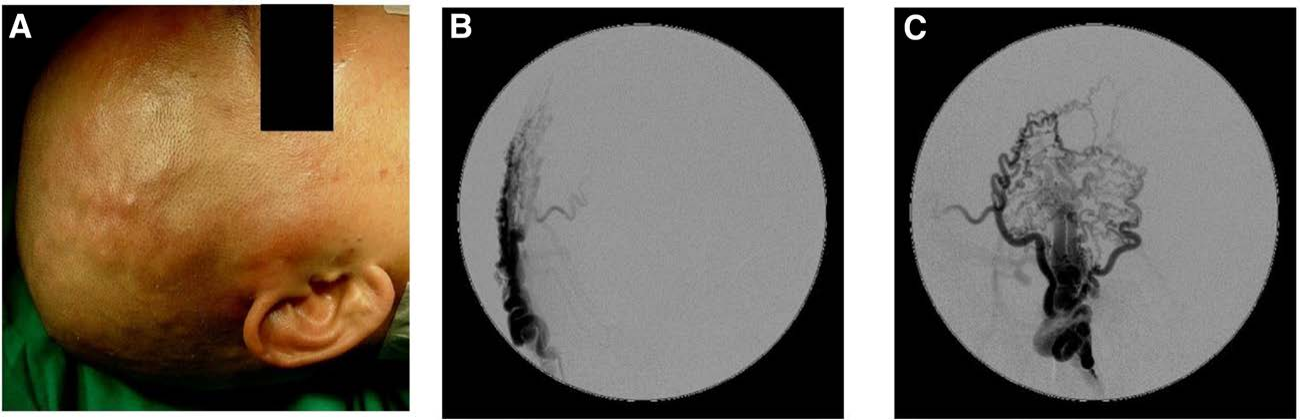
(A) Engorged and tortuous vessels were noted over right temporal area. (B, C) Preoperative brain angiography revealed scalp AVF which was fed mainly by right superficial temporal artery and drained through right superficial temporal vein. AVF = arteriovenous fistula.

**Figure 2.**
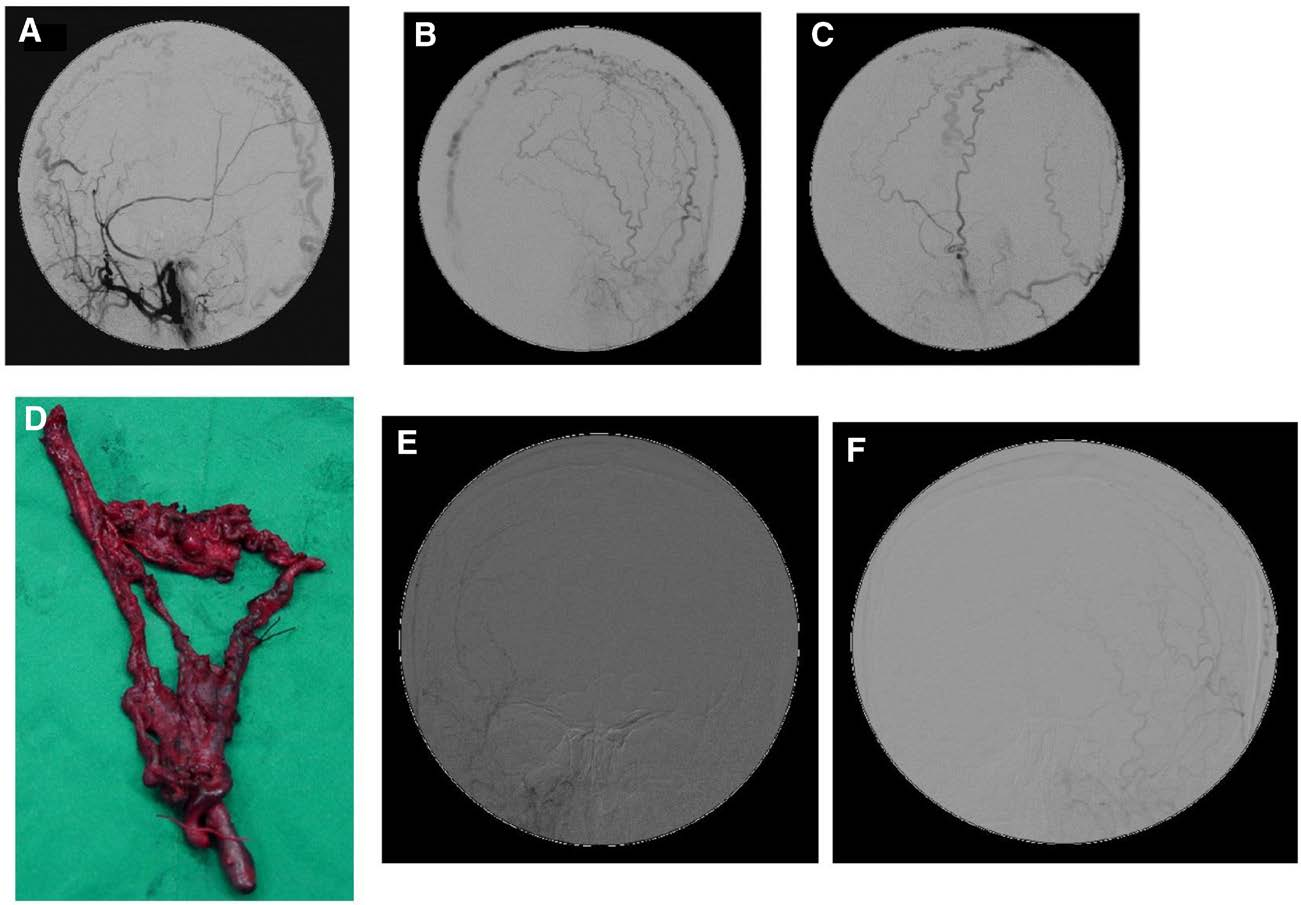
Brain angiography after trans-arterial embolization and post-OP following-up angiography. (A) Right ECA angiography revealed most feeders were embolized and some residual arteriovenous shunting was still noted. (B, C) After trans-arterial embolization, contralateral ECA supplied the fistula. (D) The specimen of scalp arteriovenous fistula was removed. (E, F) Left ECA showed no angiographic and clinical evidence of recurrence (6 mo following up). ECA = external carotid artery.

The brain angiography demonstrated an AVF was supplied by distal branches of right superficial temporal artery (STA) and was drained mainly to right superficial temporal vein (Fig. 1). Dyspnea on exertion like high output heart failure was suspected. Trans-arterial embolization (TAE) of the feeding arteries was performed with N-butyl-2-cyanoacrylate. The obliteration of the most feeders from right STA branches was achieved after embolization. However, left external carotid artery injection shows some collateral developed from left STA and occipital arteries (Fig. 2A–C). The patient suffered from headache after TAE which was supposed to result from compromised venous outlet but incomplete obliteration of the feeding arteries.

Under general anesthesia, a scalp flap was designed according to the post-embolization angiographic image. The lesion was cauterized and dissected at its periphery and totally removed (Fig. 2D). The collateral artery was also surgically ligated on the scalp flap margin. There were no postoperative complications. Follow-up at 6 months revealed no angiographic and clinical evidence of recurrence (Fig. 2E and F).

The patient had quite concerned about contralateral lesion that occurred after trans-arterial embolization. After well explained to the patient about disease possible course and he could understand well. Surgical total resection of primary site AVF and following-up angiography showed contralateral lesion subside. He felt much better and is satisfied with this treatment course.

## 3. Discussions

The etiology of the traumatic SAVF is thought to be 2 types. In 1 type, arterial injuries result in the disruption of vasa vasorum and created a hematoma round it, which becomes a pseudoaneurysm. Thus, an AVF is formed to allow the pseudoaneurysm to rupture into the adjacent vein. The other mechanism is the laceration theory, when an artery and a vein closing each other are simultaneously injured to communicated, and AVF is formed. The pathologically divided into 2 types; cirsoid aneurysms and arteriovenous fistula in a narrow sense. The former are multiple fistulae and composed of smaller varies like vessels. In the latter type, the fistula is a single communicating fistula.

Treatment options of traumatic AVF include surgical excision, ligation of feeding vessels, endovascular embolization.^[1]^ Radical excision was regard as the best form of treatment. It is important that these vessels be completely removed because they form an important collateral supply form the other collateral artery. We found that access to the fistula was facilitated by the use of preoperative embolization. In our case, following the main feeding arteries were embolized, the collateral feeding artery from contralateral external carotid artery became dominant, and we can easily perform the total complete excision of arteriovenous fistula with limited blood loss.

Despite the presence of SAVF on 1 side, bilateral brain angiography is still warranted. New lesions may develop after embolization, as in our case. Although some studies have shown good outcomes in patients with traumatic SAVF treated solely with embolization,^[2]^ other research has demonstrated the rapid progression of AVMs in the face or scalp, affecting different anatomical regions, following trans-arterial embolization.^[3]^ Subhan et al^[4]^ also demonstrate a case of traumatic SAVF where embolization was initially used to completely occlude the feeder vessels. However, the mass remained unchanged and pulsatile after 2 weeks. Consequently, surgical resection was performed afterward.^[4]^ A single therapeutic intervention for traumatic SAVF may not be appropriate in this clinical scenario.

Due to the infrequency of SAVF cases, there is presently no universally accepted approach to its treatment. Endovascular treatment for SAVF presents several challenges: not all blood vessels can be accessed, which may result in a higher likelihood of recurrence compared to surgical resection. The feeding artery of SAVF is often tortuous and difficult to maneuver with a catheter.^[5]^ While complete surgical excision of SAVF has been shown to be curative, direct surgical resection carries risks such as significant bleeding, wound infections, skin necrosis, and cosmetic issues. On the other hand, TAE may lead to reduced wound and blood loss but could also increase the risk of recurrence compared to surgical resection. By combining both TAE and surgical resection, one can balance the advantages and disadvantages. Beginning with TAE to reduce blood flow in the SAVF feeding artery, followed by surgical resection, may decrease intraoperative blood loss and reduce the likelihood of recurrence.

## 4. Conclusion

Combining endovascular embolization with subsequent surgical excision may reduce intraoperative blood loss compared to surgery alone. Additionally, a well-designed scalp flap can be implemented based on angiography findings after embolization. However, there is limited research on SAVF and a lack of long-term follow-up studies. To improve outcomes for SAVF patients, long-term follow-up and comparative studies are necessary.

## Author contributions

**Conceptualization:** Yi-Ying Hsieh.

**Investigation:** Yi-Ying Hsieh, Zhuo-Hao Liu.

**Writing—original draft:** Yi-Ying Hsieh, Zhuo-Hao Liu.

**Writing—review & editing:** Yi-Ying Hsieh, Ching-Chang Chen, Po-Hsun Tu, Shun-Tai Yang, Zhuo-Hao Liu.

**Project administration:** Ching-Chang Chen, Zhuo-Hao Liu.

**Supervision:** Ching-Chang Chen, Zhuo-Hao Liu.

**Validation:** Ching-Chang Chen, Po-Hsun Tu.

**Data curation:** Po-Hsun Tu.

**Methodology:** Shun-Tai Yang.

**Resources:** Shun-Tai Yang, Zhuo-Hao Liu.

## References

[1] Whiteside O Monksfield P Steventon N Byrne J Burton M. Endovascular embolization of a traumatic arteriovenous fistula of the superficial temporal artery. J Laryngol Otol. 2005;119:322–4. 15949092 10.1258/0022215054020368

[2] Dabus G Pizzolato R Lin E Kreusch A Linfante I. Endovascular treatment for traumatic scalp arteriovenous fistulas: results with Onyx embolization. J Neurointerv Surg. 2014;6:405–8. 23788364 10.1136/neurintsurg-2013-010724

[3] Kim ET Lee YJ Park DW Lee SR. Arteriovenous fistula at scalp: rapid progression after embolization of contralateral facial arteriovenous malformation. Neurointervention. 2010;5:36–9.

[4] Subhan M Shah S Patel S Ramanathan A. Hybrid endovascular and surgical treatment of a traumatic scalp arteriovenous fistula. Cureus. 2023;15:e49450. 38152828 10.7759/cureus.49450 PMC10751238

[5] Yokouchi T Iwabuchi S Tomiyama A Samejima H Ogata N Goto K. Embolization of scalp AVF. Interv Neuroradiol. 1999;5:121–6. 20670552 10.1177/15910199990050S122

